# Parathyroid hormone-producing cells exist in adipose tissues surrounding the parathyroid glands in hemodialysis patients with secondary hyperparathyroidism

**DOI:** 10.1038/s41598-020-60045-y

**Published:** 2020-02-24

**Authors:** Takatoshi Kakuta, Kaichiro Sawada, Genta Kanai, Ryoko Tatsumi, Takayo Miyakogawa, Mari Ishida, Raima Nakazawa, Masafumi Fukagawa

**Affiliations:** 1 Division of Nephrology, Endocrinology and Metabolism, Department of Medicine, Tokai University Hachioji Hospital, Hachioji, Tokyo Japan; 2 Division of Nephrology, Endocrinology and Metabolism, Department of Medicine, Tokai University School of Medicine, Isehara, Kanagawa Japan

**Keywords:** Parathyroid diseases, Chronic kidney disease

## Abstract

Possible ectopic parathyroid hormone (PTH) production in adipose tissues surrounding hyperplastic parathyroid glands was examined in patients with secondary hyperparathyroidism (SHPT). *In vitro* culture of adipose tissues from 31 patients excised during parathyroidectomy showed PTH secretion in 23 (74.2%) patients. *In vitro* PTH secretion was detected in adipose tissues adhered to the parathyroid glands from 22 (71.0%) patients, in not-adhered adipose from 11 (35.5%) and in the thymus from four (28.6%) patients. Immunohistochemistry revealed colonies of PTH- and GCM2-positive cells intricately intertwined with adipocytes in excised adipose tissues prior to culture. When pieces of parathyroid parenchyma from SHPT patients were transplanted into the thyroid of immunodeficient nude rats with induced SHPT, the transplants secreted human PTH for one to three-and-half months after transplantation and expressed adipocyte markers, PPARγ2 and perilipin A, that the transplants did not express prior to transplantation. These findings indicate the importance of thoroughly removing adipose tissues surrounding the parathyroid glands when performing parathyroidectomy. We speculate that these ectopic PTH-producing cells are parathyroid parenchymal cells pushed out from the glands along with adipocyte progenitors during nodular growth of hyperplastic parenchymal cells and that these cells proliferate in SHPT, forming colonies PTH-producing cells intricately intertwined with adipocytes.

## Introduction

Secondary hyperparathyroidism (SHPT) develops as an adaptive response to the defect in the regulation of calcium, phosphorus and vitamin D concentrations. SHPT is characterized by hyperplasia of the parathyroid glands (PTGs) and overproduction of parathyroid hormone (PTH), which causes bone resorption and calcification of parenchymal tissues and blood vessels, increasing the risk of cardiovascular disease^1^. Recently developed calcimimetics have notably improved the treatment and outcome of SHPT^2–5^. However, a fraction of SHPT patients become resistance to calcimimetics and eventually require parathyroidectomy^6^.

Parathyroidectomy is frequently hindered by thickly accumulated adipose tissues around hyperplastic PTGs. A comprehensive study revealed residual levels of serum PTH in SHPT patients after total parathyroidectomy^7^. Normally, humans have four parathyroid glands localized on superior right, superior left, inferior right, and inferior left positions on the thyroid. The extent of parathyroid hyperplasia in SHPT varies among the four glands even in one patient-.

Here we report the presence of PTH-secreting cells in adipose tissues surrounding PTGs in SHPT patients.

## Materials and Methods

This study adhered to the principles of the Declaration of Helsinki and was approved by the Institutional Review Board of Tokai University School of Medicine (Permission number: 14R187). Human materials were with the patients’ informed consent. Animal experimental protocols were approved by the Institutional Animal Care and Use Committee (Permission number: 171076 and 182024) and carried out according to the Tokai University Animal Experimentation Regulations.

### Human materials

The study materials were collected from 31 hemodialysis patients who underwent parathyroidectomy for severe SHPT at Tokai University Hospital. The indication for parathyroidectomy was based on the Japanese Society for Dialysis Therapy guidelines^8^. Parathyroid glands and surrounding adipose tissues were removed during parathyroidectomy and used in this study. Two types of surrounding adipose tissues were distinguishable and isolated: those that adhered tightly to and were recovered with the gland (“adhered adipose”) and others that did not adhere to but surrounded the glands (“not-adhered adipose”).

### Tissue culture

Parathyroid glands, and adhered and not-adhered adipose tissues were used in tissue culture. Specimens were also collected from the thymus and subcutaneous adipose tissues in the neck region. Tissues were weighted, minced to a size of about 5 mm^3^ by scissor, rinsed with phosphate-buffered saline (PBS), and cultured in DMEM/F12 + GlutaMAX-1 medium (Invitrogen, Carlsbad, California, USA) supplemented with 10% fetal calf serum, 50 U/ml penicillin and 50 mg/ml streptomycin in tissue culture-treated plates in a humidified atmosphere of 95% air/5% CO_2_ at 37 °C. After 24 hours, 1 ml of culture medium was collected, and intact PTH (iPTH) concentration was determined using an electrochemiluminescence immunoassay (Elecsys intact PTH; F. Hoffmann-La Roche, Basel, Switzerland) The amount of secreted iPTH (ng/day/0.1 g tissue) was calculated, and specimens that produced iPTH > 0.5 ng/day/0.1 g tissue were subjected to histological analysis.

### Immunohistochemistry

Specimens were fixed and embedded in paraffin, and 4-μm thick sections were prepared. Sections were deparaffinized, dehydrated and soaked in 1% blocking reagent (Invitrogen, Carlsbad, California, USA) in PBS. For detection of PTH- and GCM2-expressing cells, rabbit polyclonal anti-PTH antibody (sc-28922; Santa Cruz Biotechnology, Santa Cruz, CA, USA) and rabbit polyclonal ant-GCM2 antibody (ab201170; Abcam, Cambridge, UK) were used, respectively. After washing with PBS containing 0.05% Tween 20, horseradish peroxidase-conjugated anti-rabbit IgG antibody was applied. After washing as above, bound antibodies were visualized with 3,3-diaminobenzidine.

### Transplantation

Six-week-old male nude-rats (F344/NJcl-rnu/rnu; CLEA Japan Inc., Tokyo, Japan) underwent 5/6 nephrectomy as described^9^, and a few pieces of parathyroid parenchymal tissues from SHPT patients were transplanted between the thyroid and trachea. Rats were fed normal diet for a week after transplantation and then phosphate-rich pellets containing 1.2% phosphate (CLEA Japan Inc.) until the end of the experiment. Blood was sampled every week. Human iPTH concentration in the blood was determined as described above, and rat iPTH was determined with Bioactive Intact PTH ELISA kit (Immutopics International, San Clemente, CA). One to three-and-half months after transplantation, the thyroids were recovered from rats, and paraffin sections were prepared as described above for immunohistochemistry. Mouse monoclonal anti-human cytoplasm antibody (STEM121; Takara Bio Inc., Shiga, Japan; previously published as SC 121 from StemCells, Inc., Newark, CA, USA, and has been used to identify human cells in xenograft experiments^10^) was used for human tissue detection, and rabbit polyclonal anti-PPARγ2 antibody (GTX114668; GeneTex, San Antonio, TX, USA) and rabbit polyclonal anti-perilipin A antibody (ab3526; Abcam) were used for adipocyte detection. Alexa 594-conjugated goat polyclonal anti-mouse IgG antibody (ab150120; Abcam) and Alexa 488-conjugated goat polyclonal anti-rabbit IgG antibody (ab150081; Abcam) were used as secondary antibodies. Nuclei were stained with 4’,6-diamidino-2-phenylindole. Fluorescent imaging was performed with Axio Imager M2 fluorescent microscope (Zeiss, Oberkochen, Germany). Transplantation experiments were performed three times separately with parathyroid parenchymal tissues from three SHPT patients using nine to 10 rats in each experiment.

### Statistics

Data are presented as mean ± SD. Student’s *t*-test was used to compare data. Fisher’s exact test was used to examine the significance of the association between different groups. *P* values < 0.05 were considered significant.

## Results

### Ectopic PTH production in adipose tissues in the vicinity of parathyroid glands

**Figure 1:**
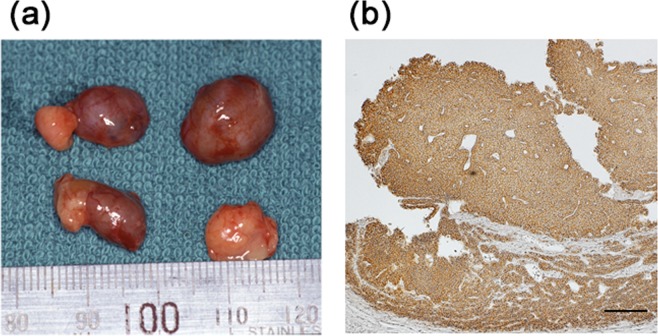
Hyperplastic parathyroid glands removed from hemodialysis patients with secondary hyperparathyroidism. **(a)** representative hyperplastic parathyroid glands and surrounding adipose tissues removed during parathyroidectomy. Two glands (upper left and lower left) have tightly adhered adipose tissues. One gland (upper right) has no adhered adipose tissues. One gland (lower right) is covered by adipose tissues. These adipose tissues did not adhere to the gland and were removed without mechanical manipulations. The scale of the ruler is a millimeter. **(b)** a representative immunohistochemical staining for PTH of a hyperplastic parathyroid gland. Bar, 200 μm.

PTGs and surrounding adipose tissues were resected from 31 SHPT patients. Two types of surrounding adipose tissues were distinguishable (Fig. 1a). One type was those that tightly adhered to PTGs and recovered with PTGs (“adhered adipose”). Staining for PTH revealed parathyroid parenchyma with adhered adipose tissues (Fig. 1b). The other type was adipose tissues that surrounded PTGs but did not adhere to the glands and were not recovered with PTGs (“not-adhered adipose”) (Fig. 1a, lower right). Not-adhered adipose tissues were collected separately from PTGs and adhered adipose tissues.

Adhered and not-adhered adipose tissues were cultured *in vitro* to determine whether they can secrete PTH. Weights of the excised parathyroid glands and adipose tissues are shown in Supplementary Table S1. An iPTH production > 0.5 ng/day/0.1 g tissue was found in 23 (74.2%) of 31 patients (Table 1). Forty-six (82.1%) of 56 adhered adipose specimens from 22 (71.0%) patients and 13 (24.1%) of 54 not-adhered adipose specimens from 11 (35.5%) patients secreted iPTH. The rate of iPTH secretion was 29.3 ± 63.7 and 6.1 ± 9.5 ng/day/0.1 g tissue in adhered and not-adhered adipose, respectively, as opposed to 125.8 ± 156.6 ng/day/0.1 g tissue in parathyroid parenchyma. Ectopic iPTH production was also found in four (28.6%) of 14 thymus specimens from 14 patients. None of the subcutaneous adipose specimens from 21 patients produced iPTH in culture. Statistical analysis showed that the patients who had PTH-secreting cells in not-adhered adipose also had these cells in adhered adipose (*P* = 0.03).

**Table 1:** PTH secretion *in vitro* by excised parathyroid parenchyma and adipose tissues.

Tissue	Detection of PTH secretion		Rate of PTH secretion (ng/day/0.1 g tissue)
	Specimens	Patients	
Parathyroid parenchyma	100% (76/76)	100% (31/31)	125.8 ± 156.6
Adipose tissues			
Adhered	82.1% (46/56)	71.0% (22/31)	29.3 ± 63.7
Not-adhered	24.1% (13/54)	35.5% (11/31)	6.1 ± 9.5
Thymus	28.6% (4/14)	12.9% (4/31)	5.9 ± 5.8
Ectopic (adipose + thymus)		74.2% (23/31)	
Subcutaneous adipose	0% (0/21)	0% (0/31)	n.d.

An iPTH secretion rate > 0.5  ng/day/0.1  g tissue was regarded as positive for PTH secretion *in vitro*. Detection of PTH secretion shows percentages of specimens or patients positive for PTH secretion *in vitro* and, in parentheses, the number of specimens or patients positive for PTH secretion *in vitro* over the number of total specimens or patients examined. Rate of PTH secretion is presented as mean ± SD. n.d., not detected.

Neither the rate of iPTH secretion nor the percentage of iPTH secreting specimens was significantly different among the four glands, except that the percentage of iPTH secreting specimens was more than twice higher for the not-adhered adipose from the superior right glands than those from other three glands (Table 2).

**Table 2:** Rate of PTH secretion *in vitro* according to gland locations.

Gland location	Parathyroid parenchyma	Adipose tissues	
		Adhered	Not-adhered
Superior right	148.0 ± 116.2 (100%, 17/17)	22.0 ± 23.1 (85.7%, 12/14)	8.6 ± 12.3 (50%, 5/10)
Superior left	128.7 ± 113.6 (100%, 19/19)	42.1 ± 114.2 (76.9%, 10/13)	7.6 ± 9.7 (23.1%, 3/13)
Inferior right	116.1 ± 136.4 (100%, 22/22)	33.6 ± 43.7 (76.5%,13/17)	1.2 ± 0.6 (23.1%, 3/13)
Inferior left	113.7 ± 231.8 (100%, 18/18)	20.5 ± 44.3 (91.7%, 11/12)	4.5 ± 3.6 (11.1%, 2/18)

Shown are the rate of iPTH secretion *in vitro* in ng/day/0.1 g tissue (mean ± SD) and, in parentheses, percentages of specimens with iPTH secretion > 0.5 ng/day/0.1 g tissue and the number of specimens with iPTH secretion > 0.5 ng/day/0.1 g tissue over the number of specimens examined.

Patient medications prior to parathyroidectomy and *in vitro* PTH secretion of resected tissues are summarized in Supplementary Table S2. There were no significant associations between medication types and *in vitro* PTH secretion.

### Detection of PTH and GCM2-positive cells in adipose tissues and the thymus

**Figure 2:**
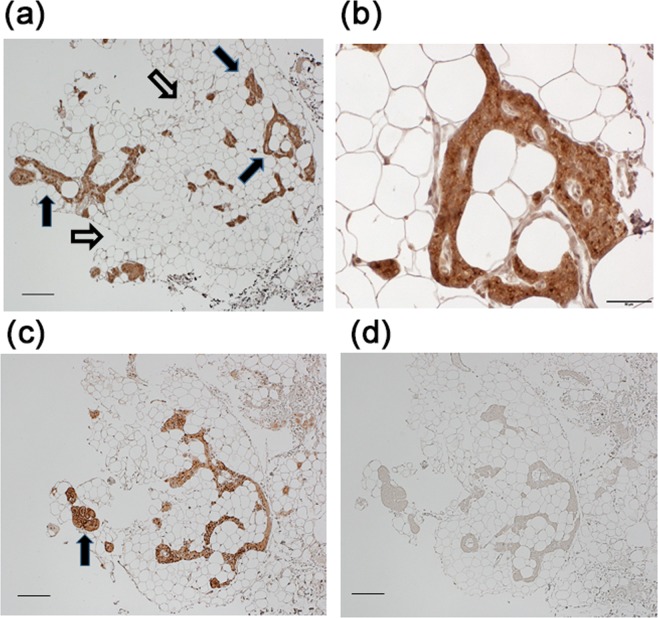
Immunohistochemistry for PTH-expressing cells in adipose tissues surrounding the parathyroid glands of secondary hyperparathyroidism patients. Adipose tissues surrounding the parathyroid glands of secondary hyperparathyroidism patients were stained with anti-PTH or anti-GCM2 antibody. Nuclei were stained with hematoxylin. Representative specimens are shown. Filled arrows indicate colonies of PTH- or GCM2-expressing cells. Empty arrows indicate adipocytes. **(a)** stained with anti-PTH antibody. A low-magnification view. Bar, 200 μm. **(b)** an enlarged view of a part of (**a**). Bar, 50 μm. **(c)** stained with anti-GCM2 antibody. A low-magnification view. Bar, 200 μm. **(d)** a negative control stained without primary antibodies.

Immunohistochemistry revealed PTH- and GCM2-positive cells in the excised adipose tissues surrounding hyperplastic PTGs prior to culture (Fig. 2). These PTH- and GCM2-positive cells had morphological features of parathyroid parenchymal cells, such as its small size and a large nucleus-to-cytoplasm ratio, were all found in the form of colonies and particularly abundant in adhered adipose tissues. These colonies of PTH- and GCM2-positive cells were intricately intertwined with adipose cells, and blood capillaries were seen supplying the colonies (Fig. 2b).

### Transplantation of parathyroid parenchyma from SHPT patients into nude rats

**Figure 3:**
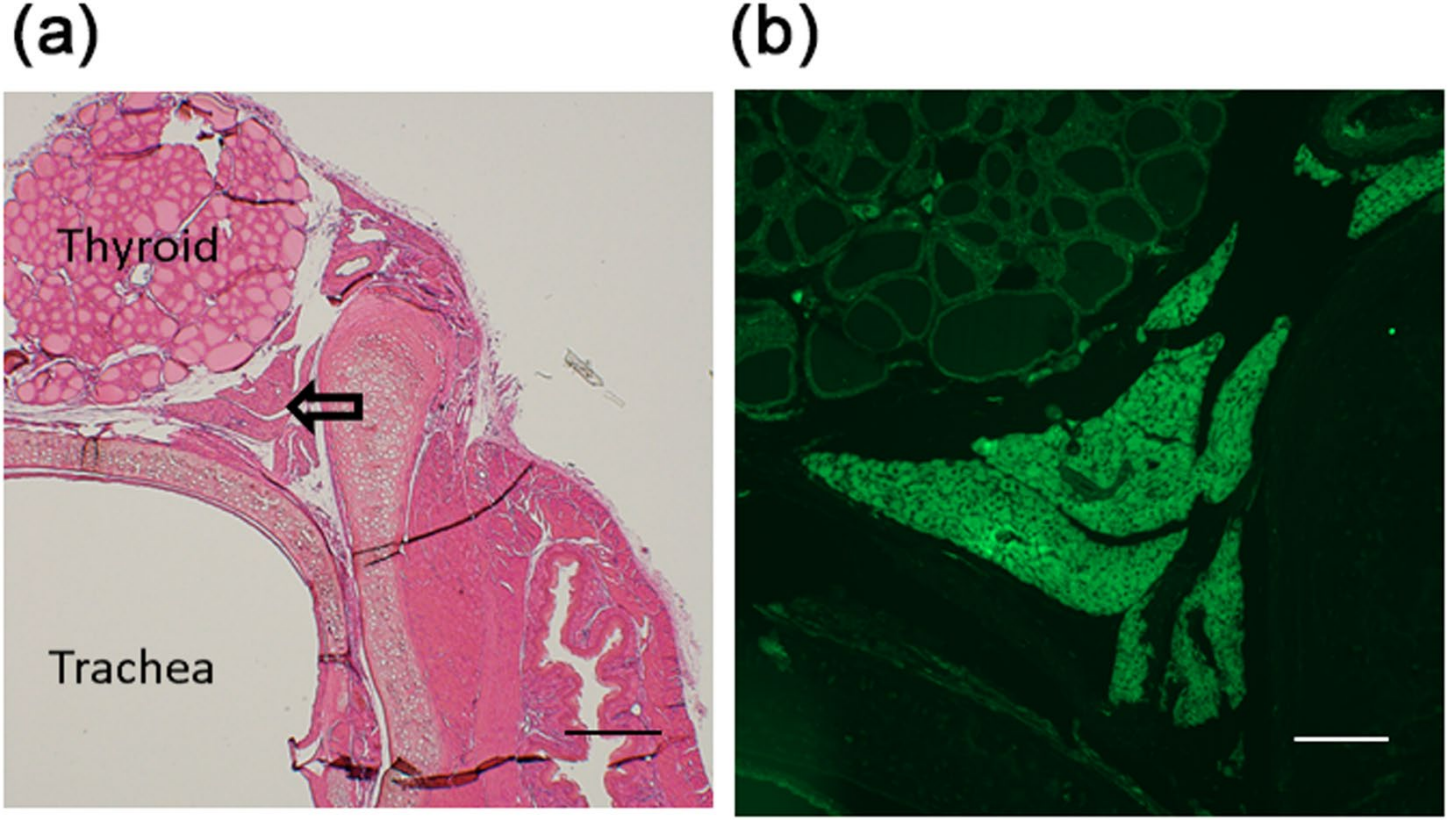
Parathyroid parenchymal tissues from secondary hyperparathyroidism patients transplanted into immunodeficient nude rats. Parathyroid parenchymal tissues from secondary hyperparathyroidism patients were transplanted between the thyroid and trachea of nude rats and recovered one to three-and-half months post-transplant. A representative specimen recovered one-month post-transplant is shown. **(a)** hematoxylin and eosin staining of human parathyroid transplants and recipient rat tissues. The empty arrow indicates human parathyroid transplants. A low-magnification view. Bar, 500 μm. **(b)** an enlarged view of a part of (**a**) stained for PTH and visualized with green fluorescence. Bar, 200 μm.

**Figure 4:**
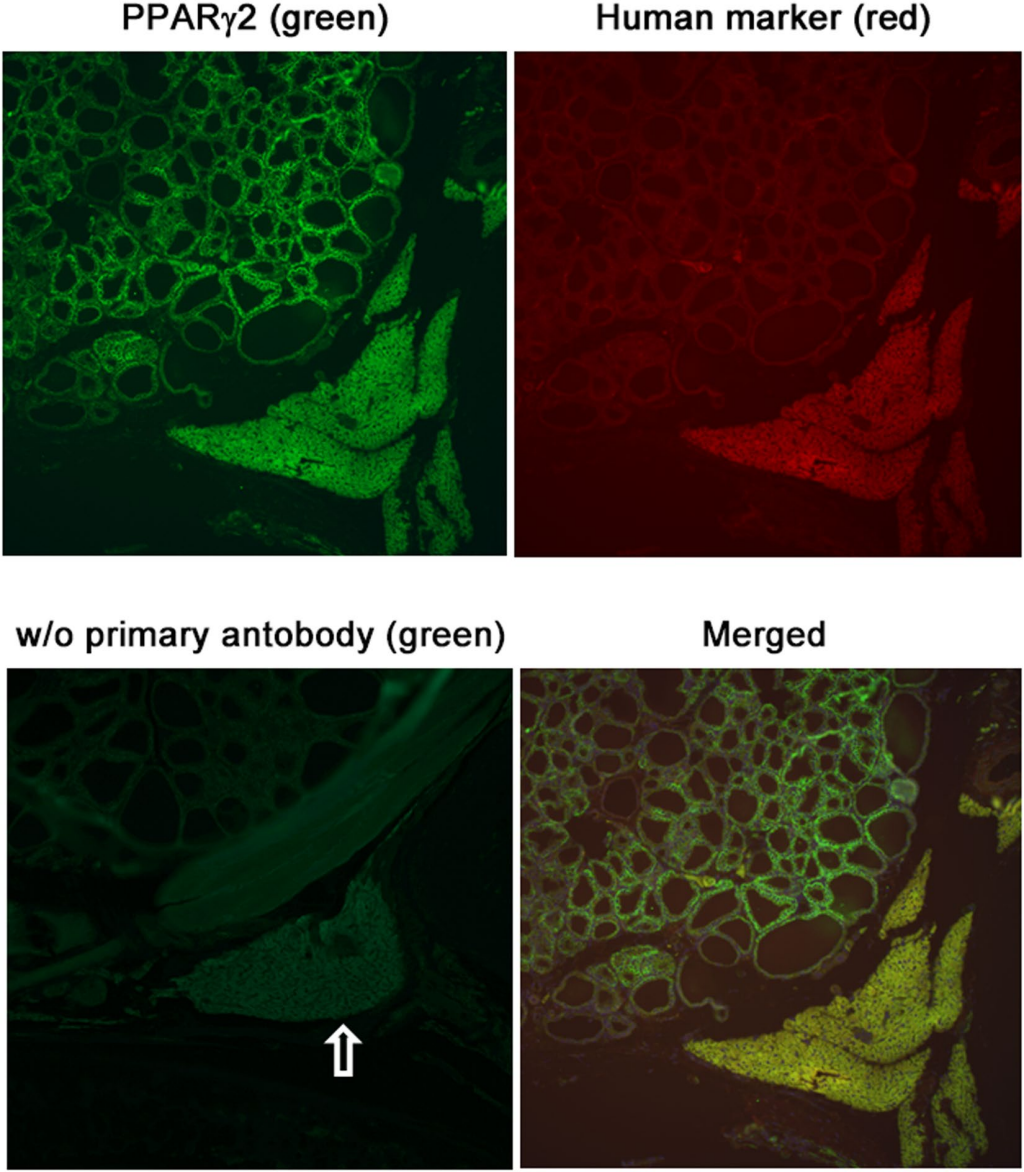
Post-transplant expression of PPARγ2 in transplanted parathyroid parenchymal tissues from secondary hyperparathyroidism patients. Parathyroid parenchymal tissues from secondary hyperparathyroidism patients transplanted into nude rats were recovered one to three-and-half months post-transplant and examined for PPARγ2 expression. A representative specimen recovered one-month post-transplant is shown. Bound anti-PPARγ2 antibody was fluorescently visualized with Alexa 488 (green) and bound anti-human cytoplasm antibody with Alexa 594 (red). These views were merged and are shown under *Merged*. As a negative staining control, the section was stained without the primary antibody and viewed with green fluorescence. The empty arrow indicates human transplants.

**Figure 5:**
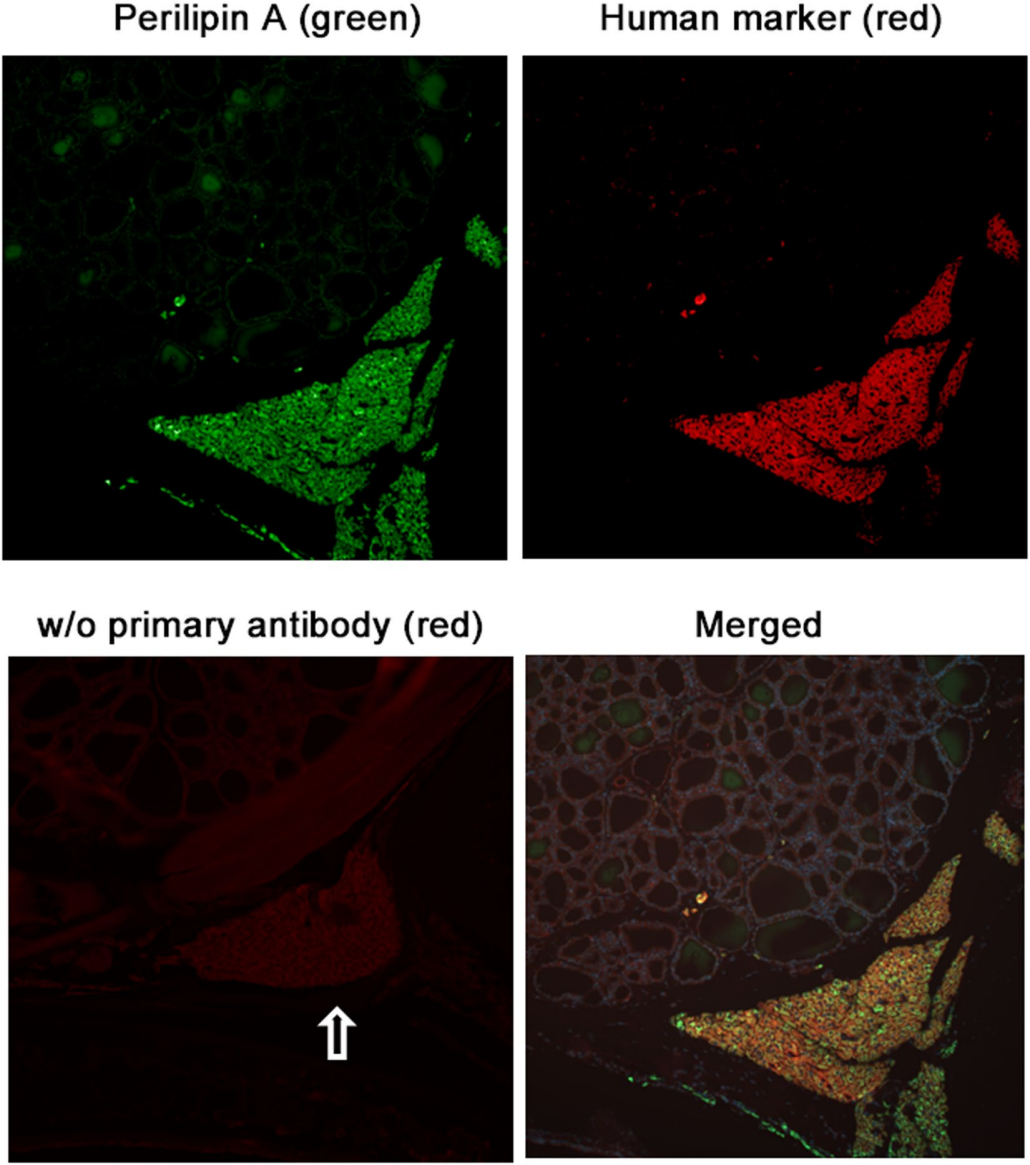
Post-transplant expression of perilipin A in transplanted parathyroid parenchymal tissues from secondary hyperparathyroidism patients. The transplants shown in Fig. 4 were examined for perilipin A expression. Bound anti-perilipin A antibody was fluorescently visualized with Alexa 488 (green) and bound anti-human cytoplasm antibody with Alexa 594 (red). These views were merged and are shown under *Merged*. As a negative staining control, the section was stained without the primary antibody and viewed with red fluorescence. The empty arrow indicates human transplants.

To investigate the relationship between ectopically located PTH-producing cells and the accumulation of adipose tissues, pieces of parathyroid parenchyma from SHPT patients were transplanted between the thyroid and trachea of immunodeficient nude rats (Fig. 3). One to three-and-half months after transplantation, transplants were recovered from rats, and analyzed for the expression of adipocyte markers. Human cells were identified using anti-human cytoplasm antibody. Prior to transplantation, the parenchyma of 12 PTGs were examined for the expression of two adipose markers, PPARγ2 or perilipin A. Neither marker was expressed in all specimens, except for one that expressed perilipin A (Table 3). However, when recovered one to three-and-half months after transplantation, all eight transplants from three SHPT patients expressed PPARγ2 (Fig. 4) and perilipin A (Fig. 5) (Table 3). Staining of the stored transplant specimens verified that they did not express either adipocyte marker prior to transplantation. All subcutaneous adipose transplants were stained positive for PPARγ2 and perilipin A (Table 3).

**Table 3:** Expression of adipocyte markers in transplants before and after transplantation.

	PPARγ	Perilipin A	PTH
Before transplantation			
Parathyroid parenchyma	0/12 (0%)	1/12 (8.3%)	12/12 (100%)
After transplantation			
Parathyroid parenchyma	8/8 (100%)	8/8 (100%)	8/8 (100%)
Subcutaneous adipose	4/4 (100%)	4/4 (100%)	0/4 (0%)

Expression of adipocyte markers, PPARγ and perilipin A, and PTH was examined in transplants by immunohistochemistry before transplantation and one to three-and-half months after transplantation. Shown are the number of specimens with expression over the number of specimens examined and the percentages of specimens with expression in parentheses. Transplants were parathyroid parenchyma and subcutaneous adipose from three SHPT patients.

## Discussion

In this study, we revealed the presence of colonies of PTH-producing cells dispersed in adipose tissues surrounding hyperplastic PTGs of SHPT patients. These ectopic PTH-producing cells expressed GCM2, had morphological features of parathyroid parenchymal cells, secreted PTH *in vitro* and were most abundant in adipose tissues tightly adhered to hyperplastic PTGs, while some were found in not-adhered adipose tissues. These ectopic PTH-producing cells were not parathyroid parenchymal cells that contaminated the specimens during parathyroidectomy or tissue handling because their colonies were intricately intertwined with adipocytes.

To investigate whether ectopically located PTH-producing cells are related to the accumulation of adipose tissues, we transplanted pieces of parathyroid parenchyma from SHPT patients into the thyroid of immunodeficient rats and induced SHPT by feeding high-phosphate diet. Neither of two adipocyte markers, PPARγ2 and perilipin A, was detected in the parenchyma of PTGs prior to transplantation, except for one of 12 specimens with perilipin A expression. After transplantation, the transplanted cells secreted human iPTH, as evidenced by elevated human iPTH levels. However, the transplants expressed both PPARγ2 and perilipin A, one month after transplantation at the earliest. The microscopically identifiable cells of the transplants retained the morphology of parathyroid parenchymal cells and lacked morphological characteristics of mature adipocyte, such as large lipid droplets. Yet, the observed expression of perilipin A, a phosphoprotein that coats the surface of lipid storage droplets^11^, suggests ongoing lipid accumulation. Thus, our findings indicate that certain cells, either parathyroid parenchymal cells or other types of cell contained in the transplants, were in early stages of differentiation into adipocytes one month after transplantation at the earliest.

As for the origin of ectopic PTH-producing cells that we found in adipose tissues surrounding PTGs of SHPT patients, they are closely related to the development of parathyroid hyperplasia because the data of *in vitro* PTH secretion showed that their abundance decreased in adipose tissues that did not adhere to PTGs. Their presence in the form of colonies indicates clonal origin, indicating the likelihood that these colonies originated from a single or a few cells. The observed expression of GCM2, a transcriptional factor that plays critical roles in parathyroid development^12^ and adult parathyroid cell proliferation and maintenance^13^, suggests a close relationship between ectopic PTH-producing cells and parathyroid cells. The fact that the colonies of ectopic PTH-producing cells were dispersed in adipose tissues and intertwined with adipocytes suggests that the progenitors of the colonies were initially dispersed in adipose tissues, proliferate along with adipocytes, and finally formed colonies within adipose tissues. Adipose tissues comprise 25–40% of a normal PTG, and adipocytes are often seen in diffuse areas rather than in nodules of hyperplastic PTGs^14^, suggesting that growing nodules tend to exclude adipocytes and their progenitors. We speculate that the ectopic PTH-producing cells are parathyroid parenchymal cells that are pushed out from PTGs with adipocyte progenitors when clonal hyperplasia progress and nodules are formed in SHPT patients. The squeezed-out parenchymal cells proliferate, and adipocyte progenitors differentiate and mature, resulting in colonies of PTH-producing cells intertwined with adipocytes. Alternatively, our transplantation experiments do not deny possible trans-differentiation of squeezed-out parathyroid parenchymal cells into adipocytes.

In summary, we demonstrated the presence of colonies of ectopic PTH-producing cells in adipose tissues surrounding PTGs of SHPT patients. Their histological localization and the findings of *in vitro* culture and transplantation experiments suggest that these ectopic PTH-producing cells are parathyroid parenchymal cells that are pushed out from PTGs during parenchymal hyperplasia and nodule formation. Our findings advise that adipose tissues associated with PTGs should be removed thoroughly in parathyroidectomy.

## Supplementary information

Supplementary Tables.
