# Supplementary material for: Parathyroid hormone-producing cells exist in adipose tissues surrounding the parathyroid glands in hemodialysis patients with secondary hyperparathyroidism
Source: Sci Rep. 2020 Feb 24;10:3290. doi: 10.1038/s41598-020-60045-y (PMC7039984; doi:10.1038/s41598-020-60045-y)
Supplement: Supplementary file 1 — Supplementary Tables. [file 41598_2020_60045_MOESM1_ESM.docx]

**[Supplementary information]**

**Parathyroid hormone-producing cells exist in adipose tissues surrounding the parathyroid glands in hemodialysis patients with secondary hyperparathyroidism**

Takatoshi Kakuta^1*^, Kaichiro Sawada^2*^, Genta Kanai^2^, Ryoko Tatsumi^1^, Takayo Miyakogawa^1^, Mari Ishida^1^, Raima Nakazawa^1^, Masafumi Fukagawa^2^

^1^ Division of Nephrology, Endocrinology and Metabolism, Department of Medicine, Tokai University Hachioji Hospital, Hachioji, Tokyo, Japan.

^2^ Division of Nephrology, Endocrinology and Metabolism, Department of Medicine, Tokai University School of Medicine, Isehara, Kanagawa, Japan.

* These authors contributed equally to this work.

Correspondence to: Takatoshi Kakuta, M.D., Ph.D.

Division of Nephrology, Endocrinology and Metabolism, Department of Medicine

Tokai University Hachioji Hospital

1838 Ishikawa-machi, Hachioji, Tokyo 192-0032, Japan.

Phone: +81 42 639 1111

Fax: +81 42 639 1112

E-mail: [kakuta@is.icc.u-tokai.ac.jp](mailto:kakuta@is.icc.u-tokai.ac.jp)

**Supplementary Tables to Kakuta et al. (SREP-19-36548-T)**

**Supplementary Table S1 Weights of excised parathyroid glands and accompanying adipose tissue**

| Parathyroid gland location | PTH secretion *in vitro* | Parathyroid parenchyma | Adipose tissues | |
| --- | --- | --- | --- | --- |
|  |  |  | Adhered | Not-adhered |
| Superior right | Positive | 0.22±0.11 (17) | 0.12±0.08 (12) | 0.10±0.07 (6) |
|  | Negative | (0) | 0.57±0.28 (2) | 0.43±0.49 (5) |
| Superior left | Positive | 0.21±0.16 (19) | 0.14±0.15 (10) | 0.25±0.14 (3) |
|  | Negative | (0) | 0.19±0.04 (3) | 0.18±0.11 (10) |
| Inferior right | Positive | 0.20±0.17 (22) | 0.14±0.07 (13) | 0.14±0.01 (3)* |
|  | Negative | (0) | 0.63±0.80 (4) | 0.42±0.29 (10)# |
| Inferior left | Positive | 0.16±0.13 (18) | 0.10±0.08 (11) | 0.25±0.12 (2) |
|  | Negative | (0) | 0.09 (1) | 0.42±0.38 (16) |

Weights of excised parathyroid glands and adipose tissues are shown by the gland location and PTH secretion *in vitro*. An iPTH secretion rate >0.5 ng/day/0.1 g tissue was regarded as positive for PTH secretion *in vitro*. Shown are weights in grams (mean ± SD) and the number of specimens in parentheses. * indicates a significant difference with negative superior right glands and # indicate a significant difference with negative superior left.

**Supplementary Table S2 Medications prior to parathyroidectomy and *in vitro* PTH secretion of resected tissues**

| Medication | | Patients (*n*) | *In vitro* PTH secretion of resected tissues | | | |
| --- | --- | --- | --- | --- | --- | --- |
|  |  |  | Adhered adipose | Not-adhered adipose | Thymus | Subcutaneous adipose |
| Calcimimetics | Regpara | 20 | 14/18  (77.8) | 5/16  (31.3) | 3/10  (30.0) | 0/17  (0.0) |
|  | Parsabiv | 5 | 5/5  (100.0) | 3/4  (75.0) | 1/1  (100.0) | 0/3  (0.0) |
|  | None | 6 | 3/4  (75.0) | 3/6  (50.0) | 0/3  (0.0) | 0/4  (0.0) |
| Vitamin D  preparations or analogs | Oxarol | 13 | 11/13  (84.6) | 5/10  (50.0) | 1/5  (20.0) | 0/12  (0.0) |
|  | Rocaltrol (oral) | 6 | 4/5  (80.0) | 3/6  (50.0) | 0/3  (0.0) | 0/3  (0.0) |
|  | Rocaltrol  (i.v.) | 4 | 2/2  (100.0) | 1/2  (50.0) | 1/1  (100.0) | 0/4  (0.0) |
|  | Alfarol | 3 | 1/3  (33.3) | 1/3  (33.3) | 1/2  (50.0) | 0/3  (0.0) |
|  | None | 5 | 4/4  (100.0) | 1/5  (20.0) | 1/3  (33.3) | 0/2  (0.0) |
| Phosphate binders | Fosrenol Chewable | 15 | 12/14  (85.7) | 6/12  (50.0) | 3/7  (42.9) | 0/13  (0.0) |
|  | Riona | 10 | 7/9  (77.8) | 2/7  (28.6) | 1/6  (16.7) | 0/7  (0.0) |
|  | Caltan | 10 | 6/8  (75.0) | 3/9  (33.3) | 0/4  (0.0) | 0/7  (0.0) |
|  | Renagel | 5 | 3/4  (75.0) | 3/5  (60.0) | 0/2  (0.0) | 0/4  (0.0) |
|  | Kiklin | 3 | 3/3  (100.0) | 2/3  (66.7) | 0/1  (0.0) | 0/2  (0.0) |
|  | P-TOL Chewable | 3 | 2/3  (66.7) | 1/2  (50.0) | 0/1  (0.0) | 0/3  (0.0) |
|  | Phosblock | 1 | 1/1  (100.0) | 0/1  (0.0) | 0/0  (0.0) | 0/0  (0.0) |
|  | None | 1 | 0/0  (0.0) | 0/1  (0.0) | 0/0  (0.0) | 0/1  (0.0) |

The 31 patients, except one, were all instructed to take low-phosphate diet.

The values shown under *in vitro* PTH secretion are the number of PTH secretion positive specimens/the number of specimens examined and the percentages of PTH secretion-positive specimens in parentheses.

Fisher’s exact test showed that there were no significant associations between medication types and *in vitro* PTH secretion.
